# A Single Case Feasibility Study of Sensorimotor Rhythm Neurofeedback in Parkinson’s Disease

**DOI:** 10.3389/fnins.2021.623317

**Published:** 2021-02-04

**Authors:** Alexander J. Cook, Kristina J. Pfeifer, Peter A. Tass

**Affiliations:** Department of Neurosurgery, Stanford University School of Medicine, Stanford, CA, United States

**Keywords:** Parkinson’s disease, neurofeedback, sensorimotor rhythm, electroencephalography, beta burst

## Abstract

Electroencephalographic activity over the sensorimotor cortex has been one of the best studied targets for neurofeedback therapy. Parkinson’s disease patients display abnormal brain rhythms in the motor cortex caused by increased synchrony in the basal ganglia-cortical pathway. Few studies have examined the effects of sensorimotor-based neurofeedback therapy in humans with PD. In this pilot study, one patient, diagnosed with Parkinson’s disease 10 years prior, participated in two consecutive days of EEG neurofeedback training to increase sensorimotor rhythm (SMR) power over the motor cortex. Using a visual display connected to ongoing EEG, the patient voluntarily manipulated SMR power, and he/she was awarded with points to positively reinforce successful increases over a predefined threshold. Recorded EEG data were source localized and analyzed for the occurrence of high amplitude bursts of SMR activity as well as bursts in the beta frequency band in the precentral cortex. The rate of SMR bursts increased with each subsequent training session, while the rate of beta bursts only increased on the final session. Relative power in the beta band, a marker of PD symptom severity, decreased over the motor cortex in the later session. These results provide first evidence for the feasibility of SMR neurofeedback training as a non-invasive therapy for reducing Parkinson’s disease related activity and upregulating SMR in the human motor cortex.

## Introduction

There is an increasing interest in developing non-invasive therapies for treating Parkinson’s and other motor-related disorders (Lee and Lozano, 2018). Current gold-standard treatments include deep brain stimulation (DBS) which involves an invasive surgical procedure (Larson, 2014; Fox et al., 2018). Development of therapies that rely on non-invasive methods would be easier to implement in a clinical or at-home setting and would reduce cost to the patient and provider. Lack of verifiable clinical measures has slowed progress in this area (Geraedts et al., 2018).

Parkinson’s Disease (PD) is often characterized as an abnormal synchrony between basal ganglia and cortical areas, especially in the beta frequency range (Eusebio et al., 2009). DBS targeting the subthalamic nucleus aims to disrupt this synchrony (Wilson et al., 2011). How this subcortical activity is related to ongoing EEG measures from the scalp is less understood. Various studies have shown both an increase and a decrease in beta activity from sensorimotor areas (George et al., 2013; Cao et al., 2020). Recently, research has suggested that physiological mechanisms producing short high-amplitude bursts in the beta band are sensitive to Parkinson’s medication and correlate with symptom severity for bradykinesia and rigidity (Feingold et al., 2015; Tinkhauser et al., 2017a,b). There is evidence that the occurrence of these short bursts of activity before the initiation of movement correlate inversely with movement speed (Lofredi et al., 2019). Therefore, the dynamics of beta activity in the motor cortex offer a potential target for improving motor-related symptoms.

Neurofeedback can offer potential therapeutic benefit by training specific brain activity using classical conditioning. Patients receive feedback from their own EEG signals, usually in the form of visual or auditory cues. One well established target of neurofeedback is the sensorimotor rhythm (SMR) relating to activity over the sensorimotor cortex (Marzbani et al., 2016). This activity is shown to be associated with motor planning, initiation, and imagery (Roth et al., 1967). SMR was one of the first targets of neurofeedback shown to change because of training (Sterman, 1977). Recently, neurofeedback experiments in MPTP-induced Parkinsonian monkeys have shown that reinforcing the occurrence of short bursts of activity in the SMR frequency range, recorded from two implanted epidural sensorimotor cortex bipolar electrodes, results in a protective effect for the development of motor symptoms (Philippens et al., 2017). Monkeys were trained to voluntarily control SMR activity through positive reinforcement of spontaneously occurring SMR spindles, which led to decreased symptom severity following induction of PD. Whether reinforcement of this brain rhythm influences PD symptoms in humans has limited evidence (Thompson and Thompson, 2008).

To translate the SMR neurofeedback protocol successfully applied to Parkinsonian monkeys (Philippens et al., 2017) for clinical tests in PD patients and demonstrate feasibility, in this case study, we use an established method for rewarding increased SMR activity which has been demonstrated in healthy participants (Doppelmayr and Weber, 2011). Over two consecutive days, a single PD patient underwent multiple rounds of training, both off and on regular medication. SMR activity was recorded from electrodes on and around the sensorimotor cortex. The patient received visual feedback in the form of a constantly updating bar graph and a point system rewarding short increases of SMR activity. PD motor symptoms were measured at regular time points using the Universal Parkinson’s Disease Rating Scale subscale III (UPDRS-III). EEG was recorded during each session and analyzed offline to examine both changes in relative power spectral density (RPSD) and burst dynamics across days of training.

## Method

### Participants

One patient with idiopathic PD participated in the EEG study and an additional patient participated in a pilot of the neurofeedback protocol. The EEG patient was in his/her mid-fifties and was diagnosed with PD 10 years prior to the start of this study. Both patients signed a written informed consent form which explained the potential risks and benefits and described the procedure of the study. The patient was made aware that he/she could withdraw from the study at any time without fear of repercussion. The EEG patient was in Hoehn & Yahr stage 2 which was assessed using a 12-h dopa challenge. He/she was taking two doses of 25 mg carbidopa and 100 mg levodopa every 3–4 h.

### Procedures

The EEG patient performed two consecutive days of EEG neurofeedback. On each day, this patient arrived in the morning having withdrawn from his/her fast-acting medication for 12 h (*off condition*). Motor-related symptoms were assessed using UPDRS-III. The patient was briefed about the neurofeedback protocol, and then performed one session of EEG neurofeedback training lasting approximately 1 h. Following the training, another assessment of motor symptoms was performed while still in an off medication state. The patient was given a 1-h break where he/she was instructed to take a normal dose of two pills carbidopa/levodopa 25/100. The second session of neurofeedback was performed approximately 1 h after medication was taken and the patient felt he/she was in an on state (*on condition*). These procedures were performed again on the second day. The off then on medication procedure was used due to the patient being unable to tolerate the extended periods of unmedicated state. Also, it is important to examine whether neurofeedback training is feasible in both conditions because dopaminergic medication is thought to affect the reward system. The drawback to this method is the difficulty in separating effects of neurofeedback and medication. The pilot patient participated in the same procedure on two separate 2-day visits. However, this patient came in off medication for 24 h on the first day and remained off medication for the second day of each visit (48 h).

### EEG Acquisition

EEG was acquired using a 256-channel EEG (HydroCel GSN, EGI, Inc., Eugene, OR, United States). For neurofeedback, EEG signals were acquired from 26 electrodes including and surrounding C3 and C4 referenced to mastoid electrodes. EEG was sampled at a rate of 1,000 Hz. Five minutes of recorded data from each round of neurofeedback were used for analysis. Resting-state EEG was used for threshold finding in the neurofeedback sessions but not recorded. Data were average referenced offline and pre-processed using the EEGLAB toolbox (Delorme and Makeig, 2004) in MATLAB (Mathworks). Source signals were extracted from the ROIs precentral right and precentral left using the Desikan-Killiany atlas (Desikan et al., 2006) taking the mean over the entire region.

### Neurofeedback Protocol

**FIGURE 1:**
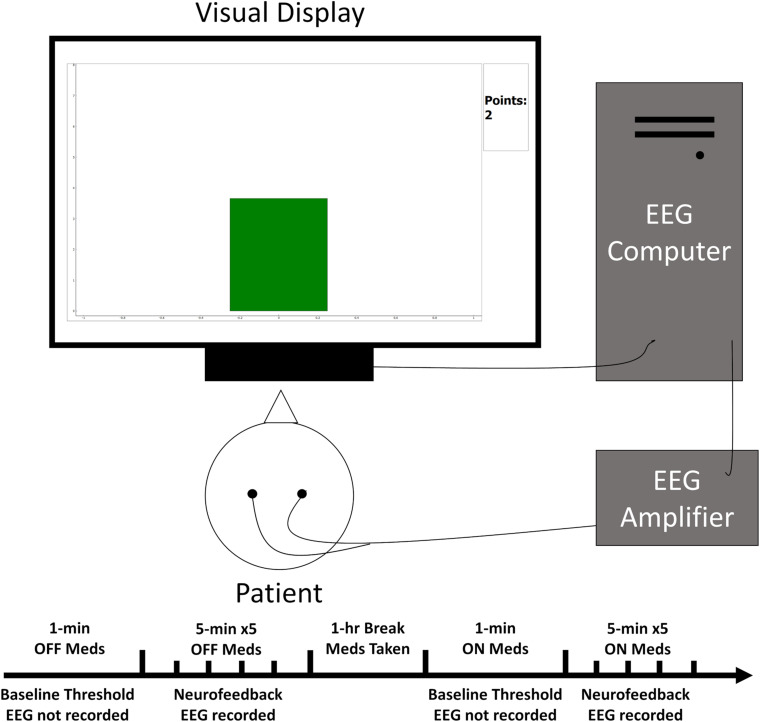
Simplified schematic of the experimental set-up. The patient sat facing the visual display while EEG electrodes relayed ongoing brain activity to the acquisition computer for processing. This activity then influenced the height and color of a bar graph on the display closing the feedback loop.

Neurofeedback training generally follows the method described in Doppelmayr and Weber (2011). Online EEG signals were processed using NeuroPype (Intheon) software. Signals were bandpass filtered for the sensorimotor frequency (12–17 Hz) used from the previous monkey study (Philippens et al., 2017), but human studies generally use 12–15 Hz (Schabus et al., 2014). Power averaged over the electrodes was calculated online to provide a single value for SMR activity (see Supplementary Methods in Supplementary Material). Muscle activity was monitored using power in motor-related frequency ranges (22–30 Hz and 45–60 Hz) and eye blinks were monitored from eye electrodes in the range 3–5 Hz. These running values were displayed using a Python script showing a single bar graph in the center of the screen that increased and decreased with the SMR value. The bar began as a neutral gray and became green for SMR and red for movement when these values were above a specified threshold described below. A point counter was displayed at the top right of the screen to provide motivation and reward of successful SMR increase (Figure 1). Before each session of neurofeedback, initial power thresholds were calculated over a 1-min resting period (see Supplementary Methods in Supplementary Material). Following thresholding, the patient was instructed to attend to the computer screen and keep movement to a minimum. The patient was instructed to try to raise a bar graph on screen until it turns green and keep it green as long and often as possible. Success in this task would be rewarded with points, which were visually displayed beside the bar graph. He/she was also told that no points were awarded when the bar would turn red indicating excessive movement. Each neurofeedback round lasted 5 min. The patient was given approximately 1 min to rest between rounds. Points were awarded if the SMR power value remained above threshold for 250 consecutive milliseconds without dropping below the threshold. The Python script was programmed such that no points were awarded for 3 s following the previous point. This assured that points would not accumulate too rapidly, which would be confusing for the patient. Thresholds were increased between rounds if the patient exceeded 55 points on the last round and decreased if he/she was awarded less than 45 points, otherwise the threshold remained constant. Thresholds were adjusted by about 3% increments, but this value was at the discretion of the experimenter to keep the patient engaged and motivated. Each session of neurofeedback consisted of five rounds.

### Relative Power Spectral Density

Power spectral density was calculated for each round in each frequency band (delta: 2–4 Hz, theta: 5–7 Hz, alpha: 8–12 Hz, SMR: 12–17 Hz, high beta: 17–29 Hz, low gamma: 30–59 Hz, high gamma: 60–90 Hz) using the Welch method. RPSD was calculated for each round in the SMR and high beta bands defined as the power in the band divided by the total power spectrum, and the values for the left and right hemispheres were averaged. RPSD was also calculated for 500 ms preceding and 500 ms following the presentation of a point to identify learning effects.

### Defining Burst Characteristics

Burst characteristics were calculated offline with the method described in Vinding et al. (2020). One threshold was used for each ROI of the source signal (see Supplementary Methods and Supplementary Figure 1 in Supplementary Material). High amplitude bursts were defined as a peak above this threshold.

Four burst characteristics were extracted from each 5-min neurofeedback round. First was the *burst rate* during the 5 min. This was used to track changes in the number of bursts across sessions of neurofeedback. The second was *burst duration*, defined as the full width at half maximum of the peak amplitude. This was used to track the time that high amplitude activity lasts. Multiple peaks that are less than one cycle apart at 12 or 17 Hz for SMR and high beta respectively were counted as a single burst. Third was the *inter-burst interval*. This was defined as the length of time between the end of one burst to the beginning of the next. The fourth measure was the *peak amplitude of the burst*.

Threshold finding and burst characteristic extraction were carried out with a custom MATLAB script. This method was repeated for both high beta (17–30 Hz) and SMR (12–17 Hz). Values were averaged across left and right hemispheres.

### Statistical Analysis

All statistical analyzes were accomplished with R statistical software (R Development Core Team, 2020). Paired, two-tailed t-tests compared day one to day two for each medication condition (on and off) separately with degrees of freedom = 4. All measures, including RPSD and all burst characteristics, were tested to discover which features of brain activity are influenced by neurofeedback training. Significant *p*-values were assessed using a critical alpha of 0.05. The effect size was assessed using Cohen’s *d*.

## Results

### Neurofeedback Performance

**FIGURE 2:**
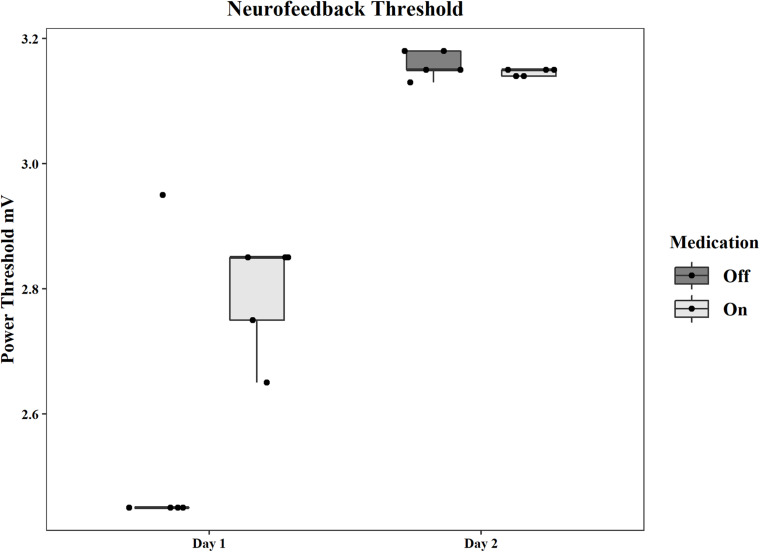
Group statistics for the power thresholds used in each neurofeedback session on each day and in each medication condition. Thresholds are in mV.

The initial average power threshold, as well as the adjusted thresholds for each subsequent round, are displayed by day and condition in Figure 2. The thresholds for the pilot patient are in Supplementary Results and Supplementary Figure 2 in Supplementary Material. Supplementary Table 1 shows all mean thresholds and mean point values for each neurofeedback round for both patients.

### Precentral Relative Power Spectral Density

RPSD was calculated for each session and each frequency band. There was no significant change in high beta power in the off condition from the first day (*M* = 0.09, *SD* = 0.02) to the second day (*M* = 0.07, *SD* = 0.01). The decrease in power in the on condition was significant (First: *M* = 0.1, *SD* = 0.002, Second: *M* = 0.07, *SD* = 0.02; *t*(4) = 4.87, *p* = 0.008, *d* = 2.18). There were no significant differences in RPSD in the SMR frequency for the off condition (First: *M* = 0.08, *SD* = 0.01, Second: *M* = 0.08, *SD* = 0.01). The decrease in the on condition was significant (First: *M* = 0.09, *SD* = 0.01, Second: *M* = 0.05, *SD* = 0.01; *t*(4) = 7.45, *p* = 0.002, *d* = 3.33). The RPSD as well as PSD for each frequency band and total PSD for all frequency bands can be seen in Supplementary Figure 3.

RPSD just before the awarding of a point was compared for the first two rounds and the last two rounds of neurofeedback for each session. The time periods immediately before and immediately after the point were also compared. However, no significant differences were found in any session of training for either comparison. Randomly selected representative EEG traces and coinciding source signals are displayed in Supplementary Figure 4.

### Burst Rate

In the off condition, the mean rate of high beta bursts on the first day was 1.04 bursts/sec (*SD* = 0.12) and 0.91 bursts/sec (*SD* = 0.10) on the second day, showing no difference. The mean rates for the on condition were 1.05 bursts/sec (*SD* = 0.19) and 3.34 bursts/sec (*SD* = 0.96) for the first and second days respectively, with a significant increase [*t*(4) = −4.64, *p* = 0.010, *d* = −2.07] from day one to day two.

**FIGURE 3:**
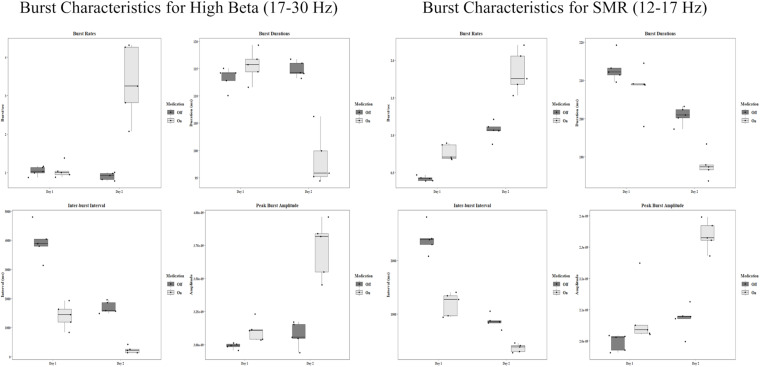
Group statistics for each day and each medication condition of all burst characteristics in the high beta band and the SMR band. Amplitude units are for sLORETA.

In the SMR band, mean rates changed in both the off condition (First: *M* = 0.42 bursts/sec, *SD* = 0.03, Second: *M* = 1.07 bursts/sec, *SD* = 0.12) and the on condition (First: *M* = 0.77 bursts/sec, *SD* = 0.11, Second: *M* = 1.84 bursts/sec, *SD* = 0.28). Both of these constitute significant increases [off: *t*(4) = −12.56, *p* < 0.001, *d* = −5.62, on: *t*(4) = −7.57, *p* = 0.002, *d* = −3.39]. The group statistics can be seen in Figure 3.

### Burst Duration

High beta burst durations show a difference only in the on condition. In the off condition the mean duration on the first day was 113.28 ms (*SD* = 1.98). On the second day, the mean was 114.89 ms (*SD* = 1.46). In the on condition the first day mean was 115.57 ms (*SD* = 2.87) and the second day mean was 98.30 ms (*SD* = 4.92). This is a large decrease in duration, *t*(4) = 9.76, *p* < 0.001, *d* = 4.37. The group statistics can be seen in Figure 3.

The SMR band shows significant decreases in burst duration from day one to day two. In the off condition, the mean on the first day was 213.15 ms (*SD* = 3.68), and the mean on the second day was 200.82 ms (*SD* = 2.28), *t*(4) = 8.15, *p* = 0.001, *d* = 3.64. In the on condition the first day mean was 207.90 ms (*SD* = 6.05), and the second day mean was 187.81 ms (*SD* = 3.54), *t*(4) = 4.70, *p* = 0.009, *d* = 2.10.

### Inter-Burst Intervals

The length between bursts for high beta showed a decrease across days. Intervals ranged from a few seconds to a few hundred milliseconds. For the off condition in the beta band the first day mean was 3,923.53 ms (*SD* = 594.58), and the second day mean was 1,691.49 ms (*SD* = 206.73), *t*(4) = 7.35, *p* = 0.002, *d* = 3.29. For the on condition, the mean on the first day was 1,406.76 ms (*SD* = 420.28) and 234.32 ms on the second day (*SD* = 115.51), *t*(4) = 5.35, *p* = 0.006, *d* = 2.39.

In the SMR band, the means for the off condition were 2,404.09 ms (*SD* = 267.05) and 861.62 ms (*SD* = 125.55) for the first and second day respectively. This is a significant decrease, *t*(4) = 16.82, *p* < 0.001, *d* = 7.52. For the on condition there was also a significant decrease from the first day (*M* = 1184.37 ms, *SD* = 219.36) to the second day (*M* = 362.92 ms, *SD* = 77.52), *t*(4) = 7.52, *p* = 0.002, *d* = 3.37. Group statistics are shown in Figure 3.

### Burst Amplitudes

Peak burst amplitudes show a general increasing pattern resembling the burst rates. In the high beta band, there was no difference between the first day (*M* = 2.99 × 10^–9^, *SD* = 2.13 × 10^–11^) and the second day (*M* = 3.07 × 10^–9^, *SD* = 9.34 × 10^–11^) for the off condition. However, there was a significant increase for the on condition from day one (*M* = 3.10 × 10^–9^, *SD* = 7.91 × 10^–9^) to day two (*M* = 3.73 × 10^–9^, *SD* = 2.15 × 10^–10^), *t*(4) = −4.88, *p* = 0.008, *d* = −2.18.

There was a slight increase in amplitude for the SMR band in the off condition [*t*(4) = −3.27, *p* = 0.031, *d* = −1.46] from day one (*M* = 2.00 × 10^–9^, *SD* = 2.65 × 10^–11^) to day two (*M* = 2.07 × 10^–9^, *SD* = 4.57 × 10^–11^). There was also a significant increase in the on condition [*t*(4) = −5.29, *p* = 0.006, *d* = −2.37] from the first day (*M* = 2.08 × 10^–9^, *SD* = 9.72 × 10^–11^) to the second day (*M* = 2.34 × 10^–9^, *SD* = 4.75 × 10^–11^). All group statistics are shown in Figure 3.

### UPDRS-III

Results of the UPDRS scoring show mixed results. On the first day, before neurofeedback a total score of 13 was recorded. After neurofeedback, the patient scored 16 while still off medication. On the second day, the patient recorded a score of 21 before neurofeedback, and a score of 16 after neurofeedback off medication. Pilot patient scores are found in Supplementary Results in Supplementary Material and all UPDRS scores are in Supplementary Table 2.

## Discussion

Burst characteristics were analyzed across sessions of reward-based SMR neurofeedback training in a single PD patient. The signal was filtered into high beta (17–30 Hz) and SMR frequency ranges (12–17 Hz) which is associated with motor inhibition and imagery. The rate of high beta bursts only increases from the first to second day when the patient is on dopaminergic medication. The same trend is seen in the peak amplitude. This increase occurs despite there being a decrease in RPSD in this frequency range. There were significant decreases in the inter-burst interval across days in both medication conditions. This suggests while bursts did not occur more frequently overall while off medication, they may occur closer together when activity becomes more “bursty.” The duration of these bursts also decreases on the second day, but only when the patient is on medication. While burst rates change dramatically on different days, the burst durations show similar ranges in all conditions, with both long and short bursts occurring despite changes in frequency. It has been shown that burst durations in the beta band are related to symptom severity, with less severe symptoms correlated with shorter durations (Tinkhauser et al., 2017b, 2018).

Burst rates in the targeted SMR frequency range also show a significant increase from the first day to the second day when the patient is on medication, and a significant increase when the patient is off medication. The trend across all four sessions of neurofeedback training shows an increasing rate of bursts. This trend was not tested for significance because of the limited scope of this feasibility study. Peak amplitudes increased for both on and off medication conditions. This occurred while RPSD in the SMR frequency range decreased in the on condition and remained constant in the off condition. The same trend in the opposite direction was seen for SMR burst duration and inter-burst intervals. Changes across days in both medication conditions show a significant decrease.

**FIGURE 4:**
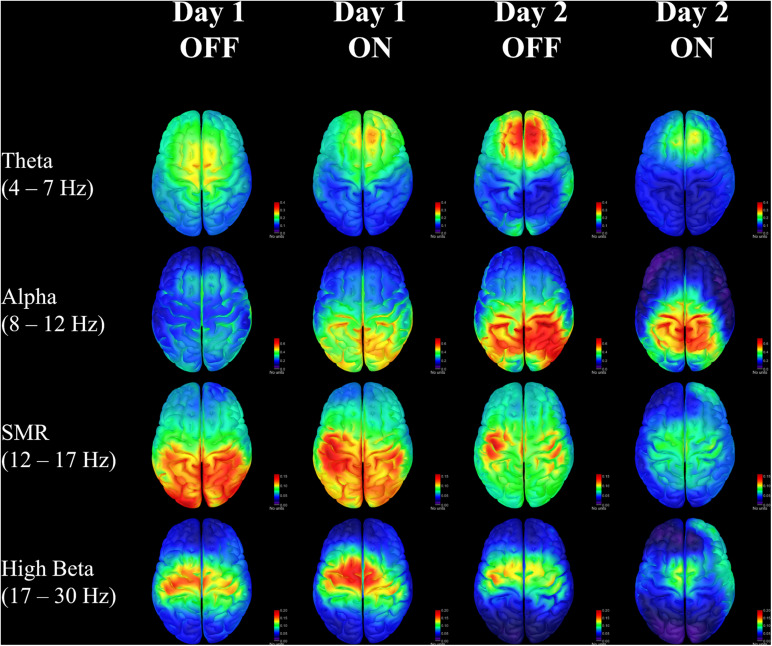
Relative power spectral density for each day and each medication condition in four frequency bands of interest. Units are for sLORETA.

The changes in the SMR band represent the most obvious findings in this case study. The neurofeedback training targeted this frequency range using scalp electrodes placed over the motor cortex. The patient was rewarded with feedback of success when bursts of SMR exceeded a threshold based on resting power for a duration of at least 250 ms. Representative traces provide visual illustration of the increasing SMR activity across training sessions. There were no significant changes in SMR power before a point was awarded from the beginning of one training round to the end of that round, suggesting learning effects build gradually over many rounds rather than occurring early on then plateauing at the end. The neurofeedback thresholds generally increased for both patients over sessions, requiring higher amplitude SMR activity to achieve feedback reward. This is reflected in the EEG results when examining burst activity in the SMR frequency range, but it is less clear whether this produced a change in non-targeted beta frequencies. However, by examining RPSD in the precentral region there appears to be a reduction of beta activity at the end of training when the patient is on medication (see Supplementary Discussion in Supplementary Material). This change becomes clearer when looking at RPSD across the entire cortex, showing diminishing dominance of beta power over the midline (Figure 4). Meanwhile, SMR power is initially distributed in posterior regions, and, while it diminishes in power over time, it becomes more dominant over the motor regions where neurofeedback is targeted.

Changes in the spatial distribution of theta and alpha RPSD also suggest that the patient may be learning cognitive control strategies to affect ongoing brain activity. There is some consensus that frontal-midline theta oscillations are involved in integrating feedback information with expected outcomes (Cavanagh and Frank, 2014; Luft, 2014), and this may be especially important for sensorimotor information in Parkinson’s (Meissner et al., 2018; Singh et al., 2020). The consolidation of theta in the frontal area seen in this patient could reflect an increased ability to monitor and respond to feedback for control of sensorimotor activity. Similarly, there is evidence that alpha activity in the right posterior hemisphere is correlated with increased internal attention (Benedek et al., 2014), which is also seen on the second day of training. While these results are related to well-known effects, it remains to be seen whether the changes occurring in one patient would be significant in a larger sample population. The patient self-reported an increased feeling of control over the neurofeedback outcome, with greater ease of predicting when successful feedback would occur. The patient was never told that a threshold change was made, however, he/she was occasionally informed that the next round would be more or less challenging. Despite this, the patient was still able to sense when a point would be awarded on more challenging rounds after some training.

The clinical significance of this approach will also need to be studied further before drawing strong conclusions. Acute changes in the UPDRS scores for each day show some mixed effects. On the second day, however, there were improvements in rigidity and gait after the first training session. From observation, the patient had severe mobility symptoms in the morning, but he/she was able to walk with moderate start and stop freezing of gait after 1 h of neurofeedback training. The short duration of the study makes it challenging to draw conclusions about the clinical measures. The scope of this study is to determine the technical feasibility and safety of neurofeedback training in Parkinson’s patients. Because neurofeedback effects are the result of long-term learning effects, it would be interesting to conduct a long-term study using neurofeedback, which should reveal whether there are stable decreases in motor symptoms over a period of weeks or months.

These results provide some initial evidence that neurofeedback targeting the SMR frequency, in analogy to the Parkinsonian monkey SMR neurofeedback training by Philippens et al. (2017), is feasible in humans with PD, both off and on medication. In future studies, computational models for burst mechanisms (Powanwe and Longtin, 2019) could be used for interpretation of findings and optimization of feedback protocols (see Supplementary Discussion in Supplementary Material). The scope of this feasibility study is limited, and a larger sample population would give more credence to these results. Sham protocols should be used to ascertain whether these changes in brain activity are a result of ongoing EEG biofeedback, or just a byproduct of the visual display and task instructions. Still, the evidence from one patient’s experience combined with the evidence in monkeys and healthy populations warrant further study of SMR neurofeedback as an adjunct therapy for PD.

## Data Availability Statement

The raw data supporting the conclusions of this article will be made available by the authors, without undue reservation.

## Ethics Statement

The studies involving human participants were reviewed and approved by Stanford University Institutional Review Board. The patients/participants provided their written informed consent to participate in this study.

## Author Contributions

AC, KP, and PT designed the study and protocol and analyzed the data. AC developed and optimized the neurofeedback setup, performed the experiments, and wrote the manuscript. PT revised the manuscript. All authors approved the manuscript.

## Conflict of Interest

The authors declare that the research was conducted in the absence of any commercial or financial relationships that could be construed as a potential conflict of interest.
