# Supplementary material for: A Single Case Feasibility Study of Sensorimotor Rhythm Neurofeedback in Parkinson’s Disease
Source: Front Neurosci. 2021 Feb 4;15:623317. doi: 10.3389/fnins.2021.623317 (PMC7890190; doi:10.3389/fnins.2021.623317)
Supplement: Supplementary file 1 [file Data_Sheet_1.PDF]

Supplementary Material

1 Supplementary Methods

1.1 Data Preprocessing and Source Estimation

Data were pre-processed in MATLAB using the EEGLAB toolbox. Individual rounds were filtered between 1 and 100 Hz with a 60 Hz notch filter. EEG data was visually inspected for bad channels. Artifact detection was automatically processed offline using the *clean_rawdata* plugin (Mullen et al., 2015) from EEGLAB. Following artifact correction, ICA was performed using the *fastica* function (Hyvarinen, 1999). Components reflecting EOG activity were removed.

Cleaned EEG was processed in brainstorm (Tadel et al., 2011) to extract source signals. A montage of 99 electrodes was selected from the 256-channel cap, providing adequate head coverage (Song et al., 2015). The same default anatomy (Fonov et al., 2011) was used for all rounds of neurofeedback, using the OpenMEEG head model (Gramfort et al., 2010). Sources were estimated using the sLORETA method (Pascual-Marqui, 2002) constrained to single dipole orientations.

1.2 Neurofeedback Protocol

Power was calculated for every sample of each EEG electrode and smoothed by averaging over the previous one second of streaming data. These power values are averaged over electrodes to obtain a single value relating to SMR power in the motor cortex which updated continuously. Before each session of neurofeedback, a one-minute baseline recording was taken. During this period, the patient was instructed to close his/her eyes for 30 seconds and open them for 30 seconds when cued by the experimenter. An average of the power was used as the initial threshold. Eye and muscle thresholds were taken as 4 times the resting eye value and 2 times the resting muscle activity values.

1.3 Burst Threshold Finding

First, the whole signal was bandpass filtered, then Hilbert transformed to extract the amplitude. A threshold was defined by x number of medians above the median amplitude of all recordings ($threshold = median + median * x$). Peaks above each threshold were counted in consecutive, non-overlapping three second windows of the data. The threshold with the highest correlation of the number of peaks to the amplitude of each window was used as the cut-off for finding peaks in the amplitude of all neurofeedback rounds.

2 Supplementary Results

2.1 Neurofeedback Thresholds

The neurofeedback threshold results for the pilot patient resemble the results of the EEG patient showing an increase across sessions. The group statistics are plotted in Supplementary Figure 2. Mean threshold amounts and point values for each session are displayed in Supplementary Table 1. Thresholds and point values were recorded for the first day of the first visit and both days of the second visit.

2.2 UPDRS-III

UPDRS-III results for the pilot patient are recorded for both days of both visits and show mixed results across days. However, there are acute decreases within days. On Day 1 of the first visit the patient had withdrawn from medication for 24 hours. The total score in the morning was 36 and the score at the end of both sessions the score was 23. On Day 2 of the first visit the patient continued to be off medication for 48 hours. The score in the morning was 30 and the score in the afternoon was 31. For the second visit, the patient had also withdrawn from medication for 24 hours and remained off medication for both days. On the first day, the total score in the morning was 20, and the afternoon score was 19. The score in the morning of Day 2 was 24, and the score in the afternoon was 23. All the UPDRS-III results are displayed in Supplementary Table 2.

3 Supplementary Discussion

Results of the power spectral density for all frequency bands combined show an increase in total power during the final session of training. It is likely that some of the burst statistics can be explained by this phenomenon, such as the burst amplitude in the high beta band. However, burst rate and burst duration are less likely to be abnormally affected by this finding. The threshold used to detect bursts considers amplitude from all the sessions, and the inclusion of this one session does not unduly inflate the value. This method of burst analysis is robust to differences between recordings of one patient, as well as between patients. It is possible this power increase is due to dyskinesia displayed by the patient. The measures of neurofeedback performance during this session, such as neurofeedback power threshold and points accumulated, remain at expected levels which further corroborates the effectiveness of this neurofeedback method in the presence of movement.

Computational models for burst generation may help to reveal the mechanisms underlying SMR bursts. For instance, in a generic model for the emergence of bursts in brain waves Powanwe and Longtin (2019) it was shown that proximity of highly synchronous dynamical regimes is associated with long bursts and short inter-burst intervals. Accordingly, a neurofeedback-induced increase of burst rate together with a decrease of burst duration might be consistent with moving the system away from highly synchronized regimes. However, so far this relationship remains speculative and requires solid experimental data achieved in a population of PD patients of sufficient size. In addition, computational results may provide hypotheses for modified neurofeedback protocols. For instance, instead of increasing SMR power one might specifically reward a decrease of burst duration and/or an increase of burst rate. On the other hand, experimental data may inform further development of computational models. For instance, the model by Powanwe and Longtin (2019) was primarily focusing on bursts in a single frequency band, e.g. gamma, thus taking into account a single population of neurons, i.e. a single brain wave. Accordingly, taking into account desired SMR bursts in combination with unwanted, PD-associated beta band oscillations might help to disentangle the impact of physiological and pathological brain oscillations.

4 Supplementary Figures and Tables

4.1 Supplementary Figures

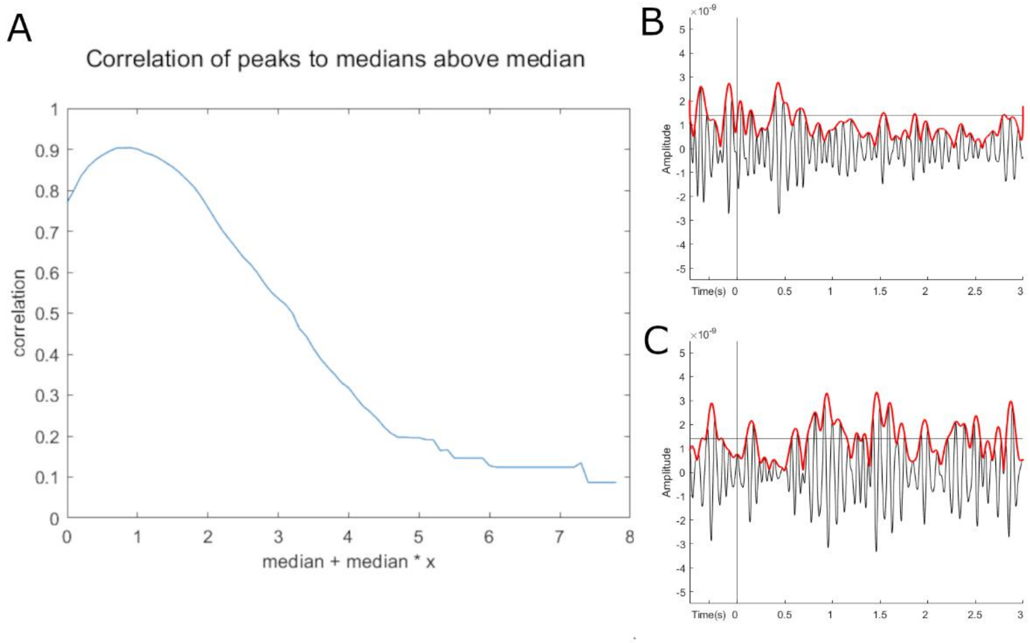

Supplementary Figure 1. (A) Shows the plot of correlation coefficients between number of peaks above the threshold to amplitude in the three-second window for each value defined as the number of medians above one median of the total amplitude of all signals in the left precentral gyrus. **(B)** Shows a representative trace from the left precentral gyrus bandpass filtered for the SMR (12-17 Hz) frequency in the Day 1 off medication condition. Red line is the Hilbert transformed amplitude envelope. Horizontal line is the threshold value with the highest correlation of number of peaks to amplitude in three-second windows. **(C)** Shows the same for Day 2 off medication. Horizontal line represents the same value threshold.

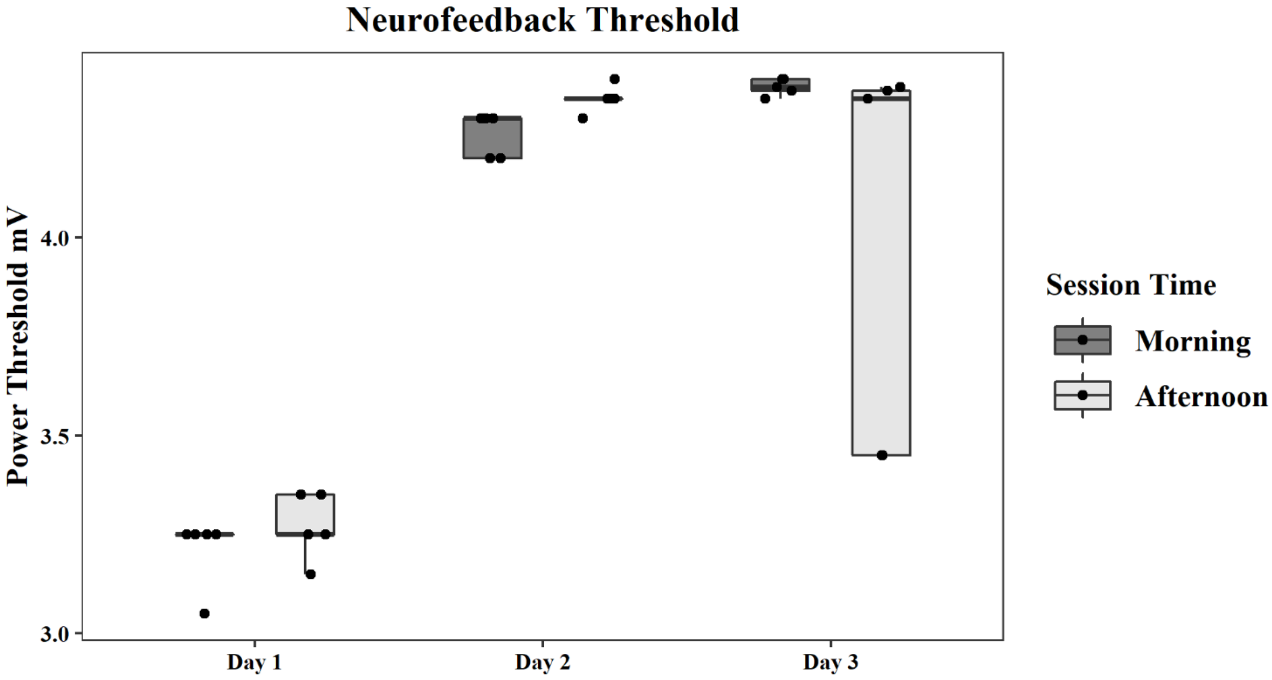

Supplementary Figure 2. Group statistics for the power thresholds used in the pilot patient. Day 1 is from the first visit. Days 2 and 3 are from the second visit. Patient is off medication during all sessions, for 24 hours in days 1 and 2 and for 48 hours in day 3.

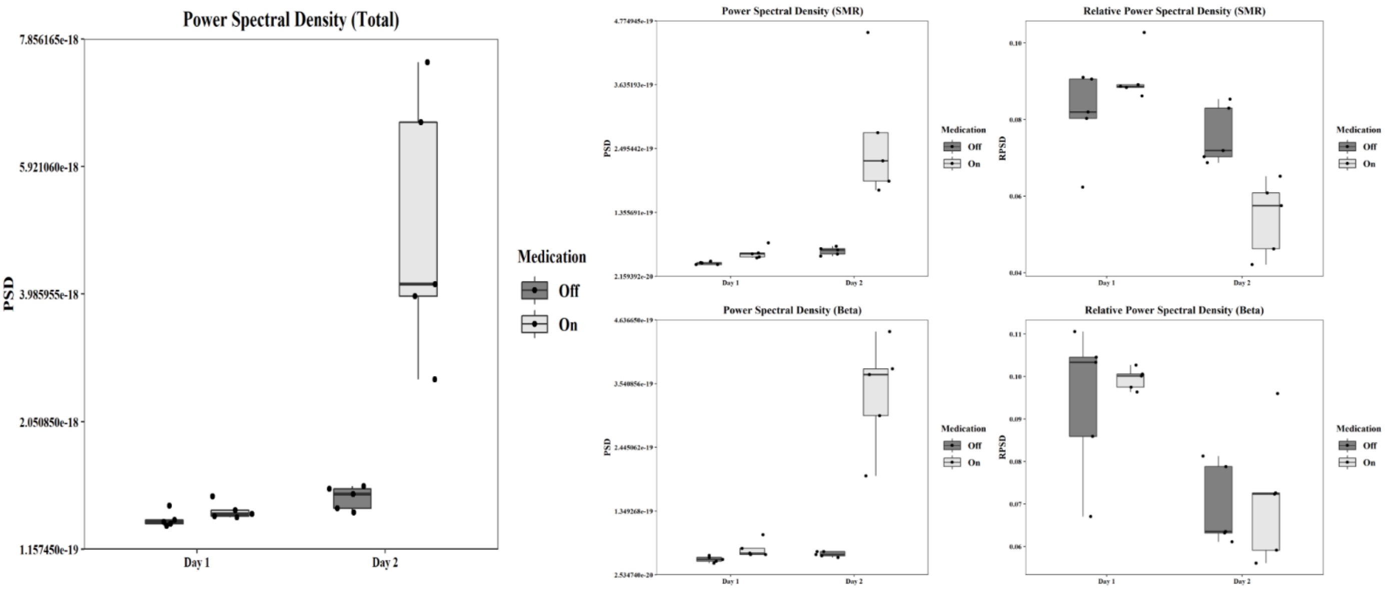

Supplementary Figure 3. Group statistics showing power spectral density (PSD) across all frequency bands for each day and each medication condition, PSD and relative power spectral density (RPSD) for SMR (12-17 Hz), and PSD and RPSD for the high beta (17-30 Hz) frequency band. Units are for sLORETA.

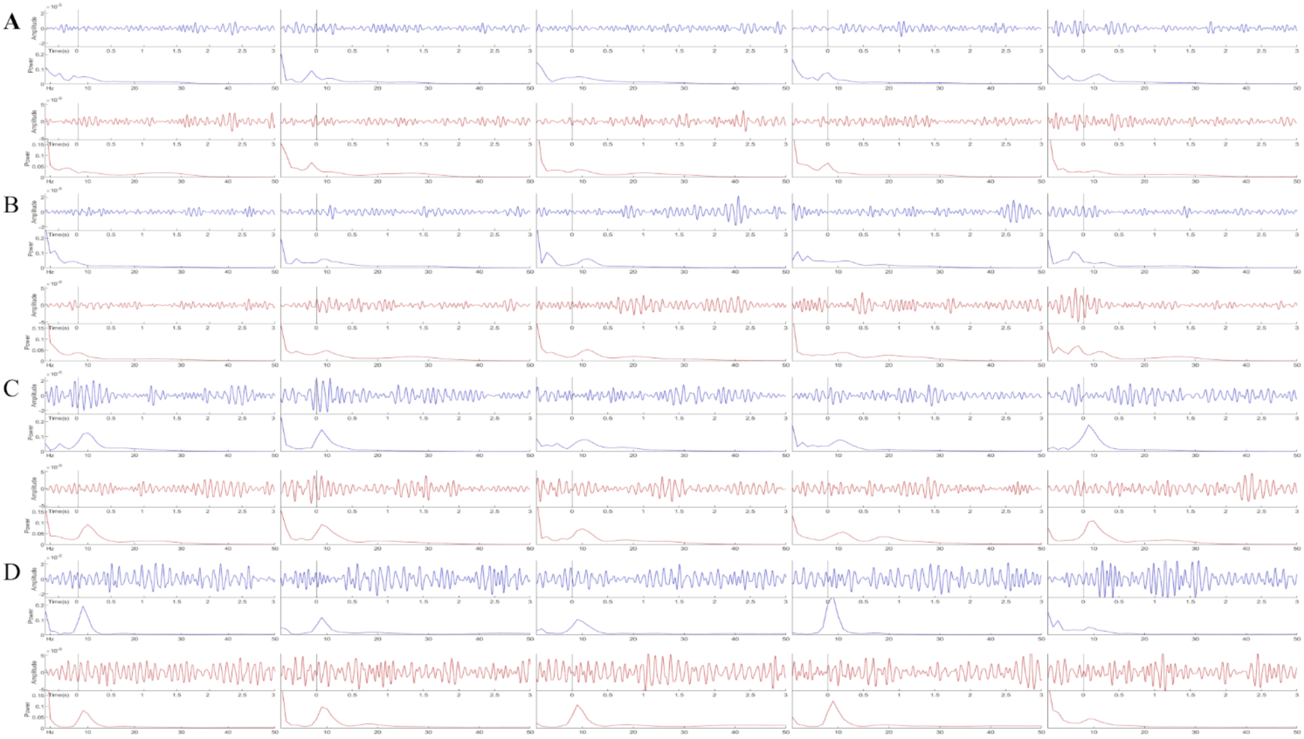

Supplementary Figure 4. Randomly selected representative traces bandpass filtered for the SMR (12-17 Hz) frequency. Blue traces are EEG recordings from electrode C3 on the left side over the motor cortex and the corresponding power spectrum. Red traces are coinciding source signals from the average of vertices located in the precentral gyrus of the left hemisphere and the corresponding power spectrum. **(A)** Day 1 off medication. **(B)** Day 1 on medication. **(C)** Day 2 off medication. **(D)** Day 2 on medication.

4.2 Supplementary Tables

	**EEG Patient**		**Pilot Patient (1st Visit)**		**Pilot Patient (2nd Visit)**	
**Session**	**Threshold**	**Points**	**Threshold**	**Points**	**Threshold**	**Points**
**1**	**2.55**	**42**	**3.21**	**52.6**	**4.26**	**50.4**
**2**	**2.79**	**44**	**3.27**	**57.6**	**4.35**	**43.4**
**3**	**3.16**	**49**			**4.38**	**42.2**
**4**	**3.15**	**41.2**			**4.00**	**39.8**

Supplementary Table 1. Total UPDRS-III scores for both days before and after the first session of neurofeedback for the EEG patient. Scores for the pilot patient on both days of both visits before the first session and after the last session of neurofeedback.

	**Day 1**		**Day 2**	
**Patient**	**Before**	**After**	**Before**	**After**
**EEG**	**13**	**16**	**21**	**16**
**Pilot (1st Visit)**	**36**	**23**	**30**	**31**
**Pilot (2nd Visit)**	**20**	**19**	**24**	**23**

Supplementary Table 2. Average threshold and average points per round for each session of neurofeedback for the EEG patient. Averages for the pilot patient on the first day of the first visit and all sessions for the second visit.
